# Piezo1 Mechanosensor Expression in Rare Hematopoietic Cells Controls Systemic Inflammatory Response in Mice

**DOI:** 10.3390/cells14241999

**Published:** 2025-12-16

**Authors:** Shiv Vardan Singh, Anastasia Iris Karkempetzaki, Nasi Huang, Vipul C. Chitalia, Saravanan Subramaniam, Katya Ravid

**Affiliations:** 1 Department of Medicine, and Whitaker Cardiovascular Institute, Boston University Chobanian & Avedisian School of Medicine, Boston, MA 02118, USA; 2 School of Medicine, University of Crete, 71003 Heraklion, Greece; 3 Renal Section, Department of Medicine, Boston University Chobanian & Avedisian School of Medicine, Boston, MA 02118, USA; 4 Department of Pharmaceutical Sciences, School of Pharmacy, Massachusetts College of Pharmacy and Health Sciences, Boston, MA 02115, USA

**Keywords:** megakaryocyte, Piezo1, inflammatory response, platelet activation

## Abstract

**Highlights:**

1. Deletion of Piezo1 in the megakaryocyte lineage does not significantly affect these cells number or ploidy, but it leads to reduced platelet activation response.
2. Piezo1 expression in hematopoietic progenitors affects white blood cell count and inflammatory response to stress.

**Abstract:**

Mutations in the Piezo1 mechanosensor are associated with blood cell anomalies. The objective of our study was to explore the role of Piezo1 in the development and function of the megakaryocyte (MK) lineage. To this end, PF4-Cre mice, bearing Cre recombinase under the control of the *Pf4* gene promoter—which drives expression to hematopoietic progenitors and to the MK/platelet lineage—were crossbred with Piezo1-floxed mice to generate Piezo1 knockout (KO) mice. In our results, the hematopoietic stem cell (HSC) count—including Multipotent Progenitors 2 (MPP2) progenitors that give rise to MKs—tended to be augmented in KO mice, while the level of MPP3 progenitors that give rise to white blood cells (WBCs) tended to be reduced, as compared to matching controls. The level of circulating WBCs was significantly reduced in the KO mice compared to controls. In addition, while platelet count was modestly elevated, platelet activation response was reduced in Piezo1 KO mice compared to controls. MK levels and ploidy were similar in both groups. Baseline serum pro-and anti-inflammatory cytokine profiles were also similar in the two experimental groups. However, upon LPS challenge, there was a significant reduction in IL-6 and INF-γ levels in the sera of Piezo1 KO mice compared to controls. Our findings point to an immunoregulatory and thrombotic potential of Piezo1 in relatively rare bone marrow cells, along with an ability to modulate WBC count.

## 1. Introduction

Mechanosensitive ion channels have been identified to play critical roles in the modulation of cellular signals, thus affecting growth, differentiation, and functions, including those of blood cells [1,2,3]. Interestingly, the growth and differentiation of hematopoietic stem and progenitor cells (HSPCs) in the bone marrow niche is mainly governed by molecular and signaling crosstalk between mechanosensors and extracellular matrix components [4,5]. Piezo1 is a cation channel that regulates cellular Ca^2+^ influx in response to forces derived from cell–cell, cell–matrix, and shear stress-mediated interactions [6,7]; this channel consists of a large three-blade propeller-shaped transmembrane protein encoded by the *FAM38A* gene, found to be activated by various extracellular stimuli, such as fluid shear stress, osmotic pressure, matrix stiffness, or cell density [7]. Additionally, Piezo1 overexpression and activation have been reported to be altered in various cell types and pathologies, thus validating its regulatory role in various cellular and physiological responses [3,8]. Piezo1-mediated Ca^2+^ signaling and activation of different kinases have been found to have regulatory effects in various cellular processes [9].

Particular to blood cell types, gain-of-function studies revealed that Piezo1 acts as a mediator for hereditary xerocytosis (HX), where dehydration of red blood cells occurs due to increased intracellular Ca^2+^ influx, thus resulting in hemolytic anemia [10]. Similarly, a retrospective study of 126 individuals diagnosed with Piezo1-HX pointed to high variability in the clinical expression of Piezo1 mutation with clinical manifestations that include anemia, splenomegaly, and post-splenectomy thrombosis [11]. Notably, functional aspects of Piezo1 are not limited to erythropoiesis since it was recently found to affect the megakaryocytic lineage also [12,13]. Herein, megakaryocytes (MKs), which are platelet progenitors, express Piezo1 and its expression has been found to be significantly upregulated in primary myelofibrosis (PMF), a disease characterized by enhanced bone marrow fibrosis and stiffness of extracellular matrix (ECM) [13]. Moreover, in human CD34+ cells and mouse MKs, Piezo1 activation has been reported to downregulate MK ploidy and platelet biogenesis [12,14]. Recently, Demagny et al. identified novel roles of Piezo1 in megakaryopoiesis, where pharmacological activation of Piezo1 induces cytosolic Ca^2+^ ion influx and reduced MK maturation (CD41+CD42+ count depleted) [12].

In a recent study, we used pharmacological approaches as well as MK-specific double-knockout (KO) Piezo1/Piezo2 mice to show the importance of this family of proteins in maintaining normal platelet levels (platelet biogenesis) [13]. In the present study, we aimed at identifying the specific roles of MK Piezo1 in determining MK and platelet levels and function using a newly developed mouse line in which Piezo1 is deleted in cells where PF4-Cre is active, particularly the MKs. Collectively, our findings point to MK/platelet Piezo1 as a direct regulator of platelet levels and activation, and surprisingly, also of some inflammatory cytokines and blood cell count profiles.

## 2. Materials and Methods

### 2.1. Animals and Transgenic Mouse Models

For the generation of PF4-Cre^+/+^Piezo1^fl/fl^ homozygous transgenic mice, C57BL/6-Tg(Pf4-icre)Q3Rsko/J mice (Jackson Laboratories), designated as PF4-Cre^+/+^, and B6.Cg-Piezo1tm2.1Apat/J mice (Jackson Laboratories), designated as Piezo1^fl/fl^, were obtained and used in this study [15]. PF4-Cre^+/+^ transgenic mice express a codon-improved Cre recombinase (iCre) under the control of mouse platelet factor 4 (*Pf4*) gene promoter. Piezo1^fl/fl^ mice have inserted LoxP sites between exons 20 and 23 of the mouse *Piezo1* gene. Two breeding cycles were performed, where from the first cycle PF4-Cre^+/−^Piezo1^fl/−^ heterozygous mice were obtained, which were further crossbred in the second cycle to generate the double-homozygous PF4-Cre^+/+^Piezo1^fl/fl^ mice (detailed in Supplementary Figure S1). In this transgenic mouse model, the *Piezo1* gene is deleted in MKs as well as in hematopoietic progenitor cells. Mouse colonies were housed at 20–22 °C on a 12 h light/12 h dark cycle, with ad libitum access to food and water. All protocols were approved by the Boston University Medical Campus Institutional Animal Care and Use Committee (IACUC).

### 2.2. Primary Mouse Bone Marrow Culture and Western Blot Analysis

Bone marrow from PF4-Cre^+/+^ and Piezo1 KO (PF4-Cre^+/+^Piezo1^fl/fl^) mice (2 males and 2 females from each genotype per each experiment, with a total of 8-10 mice analyzed) was flushed from the femurs and tibia using a 23-gauge needle and 10 mL CATCH buffer (consisting of Hank’s balanced salt solution, 0.38% sodium citrate, 1 mM adenosine, 1 mM theophylline, and 5% fetal bovine serum). Collected cells were suspended in CATCH buffer and centrifuged for 5 min at 300× *g* at 4 °C. After centrifugation, the supernatant was discarded, and cells were resuspended in 10 mL of CATCH buffer and filtered through a 100 µm cell strainer to remove any cell debris. Cells were counted using a Neubauer chamber and cultured (37 °C, 5% CO_2_) in a 6-well plate (Falcon Corning, *Cat#353046*), with a density of 10^7^ cells/mL, in IMDM media (Invitrogen, *Cat#21056*) supplemented with 10% bovine calf serum, 1000 IU/mL penicillin, and 1000 µg/mL streptomycin (Invitrogen, *Cat#15070-063*) as well as 25 ng/mL recombinant TPO (human PEG-rhMGDF, a gift from Kirin Pharma Company, Tokyo, Japan). MKs were purified after 4 days of culture, using a two-step bovine serum albumin (BSA) gradient, as described earlier [16]. Briefly, total BM cells were suspended in serum-free minimum essential medium (Thermo Fisher Scientific, *Cat#11095080*) and thereafter layered on top of a BSA gradient (upper 1.5%, and lower 3% BSA (*w*/*v*) solution) in a 15 mL conical tube. Afterwards, MKs were separated using gravity flow, then collected and rinsed twice by centrifugation with pre-warmed (37 °C) Dulbecco’s Phosphate-Buffered Saline (Thermo Fisher Scientific) for downstream application. While the purity based on cell number ranges between 50 and 70%, it is likely close to 80–90% based on MK protein and mRNA, as the gradient collects large-size, high-ploidy MKs.

For the immunoblotting of Piezo1, purified MKs were lysed for total protein extraction in radioimmunoprecipitation assay (RIPA) buffer (25 mM TrisHCl, pH 7.6, 150 mM NaCl, 1% Nonidet P-40, 1% sodium deoxycholate, 0.1% SDS) supplemented with 1× concentration of proteinase inhibitor cocktail (Thermo Fisher Scientific, *Cat#78429*) with a 30 min incubation on ice. Extracted protein was quantified using a bicinchoninic acid (BCA) assay kit (Thermo Fisher Scientific, *Cat#23225*). Moreover, a 1× concentration of SDS loading buffer was added to each sample and then normalized for an equal amount of protein in each sample. Samples were subjected to SDS-PAGE electrophoresis (4–12% polyacrylamide gel; BioRad, *Cat#4561096*), followed by transfer onto a PVDF membrane. The membrane was blocked for 1 Hr at room temperature (RT) with 5% (*w*/*v*) non-fat dry milk solution, prepared in 1× TBST (TBS buffer with 0.1% Tween−20; 20 mM Tris and 140 mM NaCl, pH 7.6) and subsequently subjected to overnight incubation at 4 °C with primary antibodies. Primary antibodies against mouse Piezo1 (Thermo Fisher Scientific, *Cat#MA5-32876*) were used in a dilution of 1:1000, whereas anti-β-actin antibodies (Sigma, *Cat#A5441*) were used in a dilution of 1:10000, respectively. Upon overnight incubation, the membrane was washed with 1× TBST and incubated for the next 1 Hr with horseradish peroxidase (HRP)-labeled anti-Mouse IgG antibodies (Cell Signaling, *Cat#7076S*) at a dilution of 1:5000. All primary and secondary antibody solutions were made in 1% bovine serum albumin (BSA) in 1× TBST. The membrane was thereafter probed with Immobilon Western Chemiluminescent HRP Substrate (Millipore, *Cat#WBKLS0100*) and bands were visualized using the chemiluminescence detection system ImageQuant LAS4000 (GE Healthcare Bio_Science Corp. Piscataway, NJ, USA).

### 2.3. mRNA Extraction and Quantitative Real-Time PCR Analysis

Total RNA was isolated from WBCs and the BSA-gradient-purified MKs, following standard protocols and using Direct-zol RNA Miniprep Kits (Zymo Research, *Cat#R2051*). A total of 1 µg RNA was used for complementary DNA (cDNA) preparation with a Quantitect Reverse Transcription cDNA kit (Qiagen, *Cat#205311*). TaqMan probes for Piezo1 (Mm01241549_m1) and GAPDH (Mm99999915_g1; housekeeping) were used as commercially available at Thermo Fisher Scientific, USA. For quantitative real-time PCR analysis, samples were run on an ViiA 7 Real-Time PCR System (Applied Biosystems, Thermo Fisher Scientific, USA). The obtained CT values were normalized to housekeeping GAPDH CT values. Using the ∆∆CT method, the relative expression of a target gene was determined by following the protocol and 2^−∆∆CT^ equation [17]. All probes were predesigned for TaqMan assays and purchased from Applied Biosystems, USA.

### 2.4. Megakaryocyte Number and Ploidy Analysis

The quantification of mature and immature MKs in BM cells isolated from PF4-Cre^+/+^ and PF4-Cre^+/+^Piezo1^fl/fl^ mice was performed using flow cytometry and the standard protocol detailed earlier [18]. As mentioned above, cells from mouse primary bone marrow cultures after day 4 were collected and rinsed twice with ice-cold 1× PBS. Cells were thereafter stained for their viability using a Zombie Aqua probe (BioLegend, *Cat#423102*) at a 1:200 dilution and incubation of 15 min at room temperature (RT). Subsequently, cells were stained with the phycoerythrin (PE)-conjugated anti-CD41 antibody (eBioscience, *Cat#12-0411-83*) and allophycocyanin (APC)-conjugated anti-CD42 antibody (Invitrogen, *Cat#17042182*) at 1:200 dilutions, with incubation for 15 min at RT. Immature MK counts were identified by gating the CD41^+^CD42^−^ cells, while mature MK counts were quantified by gating the CD41^+^CD42^+^ cells. To analyze the ploidy state of MKs in these samples, cells were rinsed with ice-cold 1× PBS and fixed with ice-cold 70% ethanol. Afterwards, cells were stained for 15 min at RT with a 1:200 dilution of FITC-conjugated anti-CD41 antibody (BD Bioscience, *Cat#553848*). Right before the analysis, RNAse A (Sigma, *Cat*#*R6513*) at 0.1 mg/mL and propidium iodide (Sigma, *Cat*#*P4170*) at 0.05 mg/mL were added to these samples, respectively. All samples were run on an LSRII flow cytometer (BD Bioscience), and data were collected using FACSDIVA^TM^ software (v9.0) and analyzed with FlowJo software version 10.8.1 (BD Biosciences, USA).

### 2.5. Peripheral Blood Count in Mice

Peripheral blood from PF4-Cre^+/+^ and PF4-Cre^+/+^Piezo1^fl/fl^ mice (males and females between 12 and 20 weeks old, n = 37 and n = 35, respectively) was withdrawn via the retro-orbital plexus bleeding method using a heparinized capillary tube under isoflurane anesthesia [19]. Blood parameters were measured in these blood samples using a Hemavet HV950FS hematology automated counter (Drew Scientific, Waterbury, CT, USA).

### 2.6. Quantification of Hematopoietic Stem Cell Population

Bone marrow from PF4-Cre^+/+^ and PF4-Cre^+/+^Piezo1^fl/fl^ mice (males and females between 15 and 25 weeks old, n = 10 and n = 12, respectively) was isolated, and samples were prepared for flow cytometric analysis, as mentioned above. For HSC populations (LSK, LT-HSC, ST-HSC, MPP2, MPP3/4, MKP, and CD41^+^) analysis, BM cells were stained with a cocktail of lineage-specific markers, viz., anti-B220 (BD Pharmingen, *Cat#552094*), anti-CD11b (BD Pharmingen, *Cat#557657*), anti-Ter119 (BD Pharmingen, *Cat#560509*), all conjugated with the same fluorochrome APC-Cy7. Further staining with the stem cell markers, viz., anti-Sca-1-PerCP-cy5.5 (Invitrogen, *Cat#45-5981-82*), anti-c-kit-BV421 (BD Pharmingen, *Cat#562609*), anti-CD150-APC (Invitrogen, *Cat#17-1502-80*), anti-CD48-PE (Invitrogen, *Cat#12-0481-82*), anti-CD41-BV510 (BD Pharmingen, *Cat#740136*), and Zombie-NIR (viability-specific), in a dilution of 1:200 for all the antibodies. Following incubation for 15 min at RT and protected from direct light, samples were analyzed on a five-laser Aurora (Cytek) flow cytometer. The collected data were analyzed with FlowJo software (version 10.8.1) from BD Biosciences, USA.

### 2.7. Inflammatory Cytokine Measurement

Baseline measurement of inflammatory cytokines in the serum samples derived from PF4-Cre^+/+^ and PF4-Cre^+/+^Piezo1^fl/fl^ mice (n = 9–14; both males and females) was measured by collecting the blood from mice using standard protocols mentioned above. Inflammatory cytokines (TNF-α; *Cat#2673KI*, IL-6; *Cat#2653KI*, and IFN-γ; *Cat#2612KI*) in the serum samples were quantified by using the commercially available mouse-specific ELISA kits, supplied from BD Biosciences, USA. Following the manufacturer’s instructions, quantification of cytokines was performed using standard curves prepared from cytokine standards supplied with the kits. Furthermore, to estimate the innate immune potential of PF4-Cre^+/+^ and PF4-Cre^+/+^Piezo1^fl/fl^ mice, mice were challenged with an intraperitoneal (i.p.) injection of lipopolysaccharide (LPS) at a dose of 0.5 mg/kg (prepared in normal saline). Blood was collected from mice through the retro-orbital plexus bleeding method, at 3 h and 24 h interval after the LPS challenge. Using standard protocols, serum was prepared and stored at −80 °C for the analysis of serum inflammatory cytokines.

Similarly, inflammatory cytokines in the BM and MK culture supernatants were also measured. BM cells were procured and cultured in vitro in similar conditions as mentioned above (Section 2.2). After the 4th day, BM cells were collected and centrifuged at 300× *g* for 5 min at 4 °C. Supernatant was collected in a separate tube and stored at −80 °C for further quantification of inflammatory cytokines. Cells were washed once with ice-cold 1× PBS and re-suspended in CATCH buffer for a two-step bovine serum albumin (BSA) gradient-based separation of MKs, as described above. Isolated MKs were counted using a Neubauer chamber (Falcon Corning, *Cat#480200*) and re-seeded in a 24-well plate, with a density of 2 × 10^4^ cells/mL in IMDM media. Following 24 h of incubation, cells were collected along with the culture supernatant and centrifuged at 300× *g* for 5 min at 4 °C to collect the culture supernatant and MKs, separately. Supernatant was used for the quantification of inflammatory cytokines, and MKs were used for the isolation of total RNA, cDNA synthesis, and mRNA expression analysis of inflammatory genes using qRT-PCR.

### 2.8. Measurement of Tail Bleeding Time

PF4-Cre^+/+^ and PF4-Cre^+/+^Piezo1^fl/fl^ mice (n = 12; both males and females) were randomly selected and anesthetized using isoflurane anesthesia, and the tail bleeding experiment was performed by following the standard protocol [20]. Mice were placed on a heat pad and 3 mm of the tail tip was cut with a scalpel and immersed immediately in saline (0.9% NaCl) at 37 °C in a 50 mL conical tube. Time for bleeding and cessation of blood stream was measured and recorded for each mouse. Absence of bleeding for 1 min was considered as complete cessation whereas total time for recording was 20 min from of the tip of the tail, including partial cessations that resume within 1 min.

### 2.9. Whole Blood Platelet Aggregation Assay

Platelet aggregation was evaluated using whole-blood impedance aggregometry (Model 700; Chrono-Log Corporation, Havertown, PA, USA), as previously described [21,22]. In brief, citrated whole blood (200 μL) was mixed with normal saline (800 μL) in a disposable cuvette (Chrono-Log Corporation, Havertown, PA, USA). An electrode was immersed in the sample, and platelet aggregation was measured based on the increase in electrical impedance (mms) caused by platelet clumping on the electrode surface. In vitro platelet activation was induced using mouse-derived collagen (5 μg/mL) and thrombin (0.025 U/mL) (Chrono-Log Corporation, PA, USA). The aggregation reaction was monitored for 6 min at 37 °C with a stirring speed of 1200 rpm. Data acquisition and analysis were performed using Aggrolink-8 software (Chrono-Log), and results are expressed as area under the curve (AUC; ohmxminute), lag time (minutes), slope, and maximum aggregation (A_max_; ohm).

### 2.10. Quantitative Expression Analysis of Inflammatory Genes

Total RNA extraction was performed by following the cDNA synthesis using an Invitrogen Thermoscript™ RT-PCR kit by following the manufacturer’s protocol. Quantitative real-time PCR was performed using the following TaqMan gene expression primers and probes, purchased from Applied Biosystems with detailed assay IDs: interleukin-6 (Mm00446190_m1), tumor necrosis factor-α (Mm00443258_m1), interferon-γ (Mm01168134_m1), and 18s rRNA (Mm04277571_s1). PCR reaction efficiency and primer concentration were optimized, and qRT-PCR was performed with a ViiA 7 RT-PCR system (Applied Biosystem, Thermo Fisher, USA). The relative expression of TNF-α, IL-6, and IFN-γ mRNA was normalized to the amount of 18sRNA in the same samples, using the relative quantification of the ∆∆CT method mentioned above. Relative expression of the target gene was expressed as the fold change in the target gene, corresponding to its internal housekeeping control.

### 2.11. Statistical Analysis

Experimental data are expressed as mean ± SE values of three experiments performed in triplicate. A two-tailed Student’s “*t*” test was used for the statistical analysis, comparing two experimental groups. One-way ANOVA was performed, followed by Tukey’s post hoc test for the comparison of a single factor in multiple groups. GraphPad Prism 8 (GraphPad Software) was used for the statistical analysis and graphing. *p* values of *** *p* < 0.001, ** *p* < 0.01, * *p* < 0.05 were considered statistically significant.

## 3. Results

### 3.1. Piezo1 Deletion in Megakaryocytes Does Not Alter These Cells’ Number or Ploidy Level

**Figure 1:**
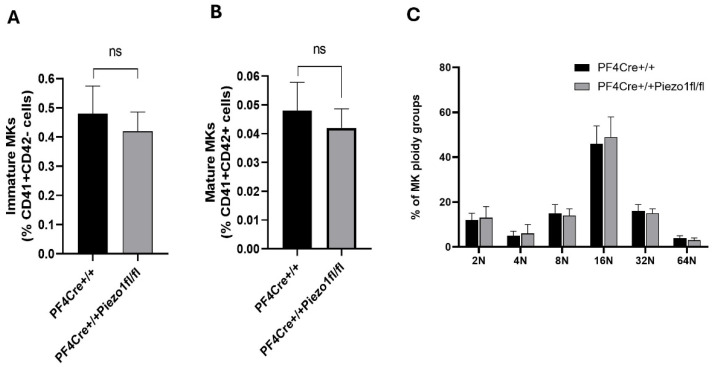
Effect of megakaryocyte (MK) Piezo 1 deletion on bone marrow MK number and ploidy. (**A**) Flow cytometry analysis of immature MKs (CD41+ and CD42d-); (**B**) mature MKs (CD41+ and CD42d+). (**C**) Flow cytometry analysis of ploidy profiles of MKs isolated from PF4-Cre^+/+^ and PF4-Cre^+/+^Piezo1^fl/fl^ mice. Data are expressed as mean ± SE of three independent experiments performed separately using 12–14-week-old male and female mice. ns: denotes no statistical difference.

To study the role of Piezo1 in megakaryopoiesis, we generated mice with Piezo1 deleted in MKs (scheme depicted in Supplementary Figure S1A) by crossbreeding Piezo1-floxed mice with mice expressing Cre recombinase under the control of a *Pf4* gene promoter. BSA gradient-purified MKs (see the Methods Section) were tested for Piezo1 expression at protein and mRNA levels. Herein, PF4-Cre mouse MKs have a clear expression of Piezo1, while MKs isolated from Piezo1 KO mice show a minimal expression level (Supplementary Figure S1B). Piezo1 expression at the mRNA level was also found to be lowered by 81.78 ± 1.15% in Piezo1-KO mice-derived MKs compared to experimental control (PF4-Cre) MKs (Supplementary Figure S1C). Residual levels of expression could be attributed to other cells contaminants, or/and to incomplete activity of Cre in all targeted cells. Afterwards, the number of mature MKs (CD41^+^/CD42^+^) and MK ploidy levels were also determined, with no significant differences found between the experimental groups (Figure 1A–C).

### 3.2. PF4-Cre-Mediated Deletion of Piezo1 Leads to Low White Blood Cell Count and a Modest Increase in Platelet Count

**Figure 2:**
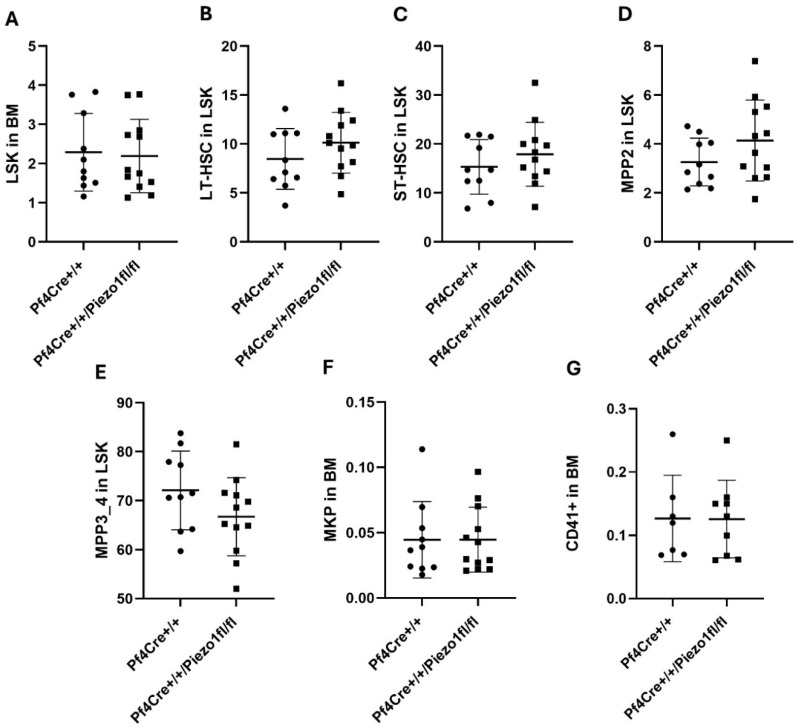
Percentage of hematopoietic stem cell (HSC) populations. Spectral flow cytometry was used to determine HSC populations in bone marrow (BM) cells isolated from 10 PF4-Cre^+/+^ and 12 PF4-Cre^+/+^Piezo1^fl/fl^ male mice, 15–25 weeks old. (**A**) The percentage of LSK cells in the BM was determined by gating Lin-Sca-1+c-kit+ cells. (**B**) Percentage of LT-HSCs in the LSK population: CD150+CD48+ cells. (**C**) Percentage of ST-HSCs in the LSK population: CD150-CD48- cells. (**D**) Percentage of MPP2 in the LSK population: CD150+CD48+ cells. (**E**) Percentage of MPP3/4 in the LSK population: CD150-CD48+ cells. (**F**) Percentage of MKP in the BM. (**G**) Lin-Sca1-c-kit+ CD41+CD150+ cells. Data are expressed as mean ± SE of three independent experiments performed separately using at time cells pooled from 3 mice per genotype. In panels (**D**,**E**), the observed up and down, respectively, trends were close to statistical significance but did not reach *p* < 0.05.

As published earlier [23], the PF4-Cre line drives expression to the megakaryocytic/platelet lineage, but also to their progeny, including Multipotent Progenitors 3 (MPP3) and MPP4. Thus, hematopoietic stem cells (HSCs) and total blood cell counts were also measured to estimate the effect of Piezo1 deletion on bone marrow hematopoiesis. Levels of LSK cells in the BM, determined by gating on Lin-Sca-1+c-Kit+ cells, were similar in Piezo1 KO mice and matching PF4-Cre mice (Figure 2A). While there was a tendency for lower numbers of LT-HSCs (long-term hematopoietic stem cells) (Figure 2B) and ST-HSCs (short-term hematopoietic stem cells) (Figure 2C) in Piezo1 KO mice, compared to age- and sex-matched PF4-Cre controls, the differences did not reach statistical significance. MPP2 count (MK progeny) and MPP3/4 (progenitor cells committed for white blood cells) also tended to be at higher and lower levels, respectively, in Piezo1 KO mice, as compared to PF4-Cre control mice (not statistically significant) (Figure 2D,E). The number of MK progenitors (MKPs) and of CD41^+^ cells was similar in the two experimental groups (Figure 2F,G).

**Figure 3:**
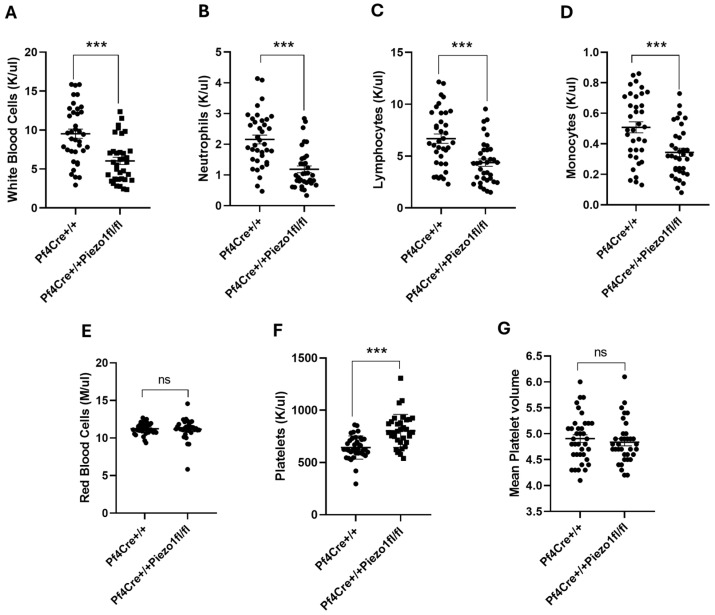
Peripheral blood cell counts in PF4-Cre and Piezo1 KO mice. Complete blood cell counts measured in PF4-Cre^+/+^ and PF4-Cre^+/+^Piezo1^fl/fl^ mice (males and females between 12 and 20 weeks old, n = 37 and n = 35, respectively, analyzed as age-matched groups). (**A**) White blood cells. (**B**) Neutrophils. (**C**) Lymphocytes. (**D**) Monocytes. (**E**) Red blood cells. (**F**) Platelets. (**G**) Mean platelet volume. Data are expressed as mean ± SE, where *** *p* < 0.001 values are considered significantly different between PF4-Cre^+/+^ and PF4-Cre^+/+^Piezo1^fl/fl^ mice. ns: denotes no statistical difference.

As to blood cell count, total white blood cell (WBC) count was found to be significantly lowered (36.36%; *p* < 0.001) in Piezo1 KO mice (Figure 3A), compared to PF4-Cre controls. Among WBCs, the levels of neutrophils (NE), lymphocytes (LY), and monocytes (MO) were all significantly (*p* < 0.001) lowered in Piezo1 KO mice (Figure 3B–D). In turn, Piezo1 mRNA expression was also estimated in the WBCs isolated from PF4-Cre and Piezo1 KO mice, where no significant difference was found between these groups (Supplementary Figure S2); this suggested that PF4-Cre is no longer active in fully differentiated WBCs. There was no significant difference in red blood cell (RBC) count between the experimental groups (Figure 3E). Platelet counts tended to be higher in the KO mice (Figure 3F), with no change in mean platelet volume (MPV) upon Piezo1 deletion (Figure 3G).

### 3.3. Megakaryocyte Piezo1 Deletion Significantly Prolongs Bleeding Time and Reduces Platelet Activation Responses

**Figure 4:**
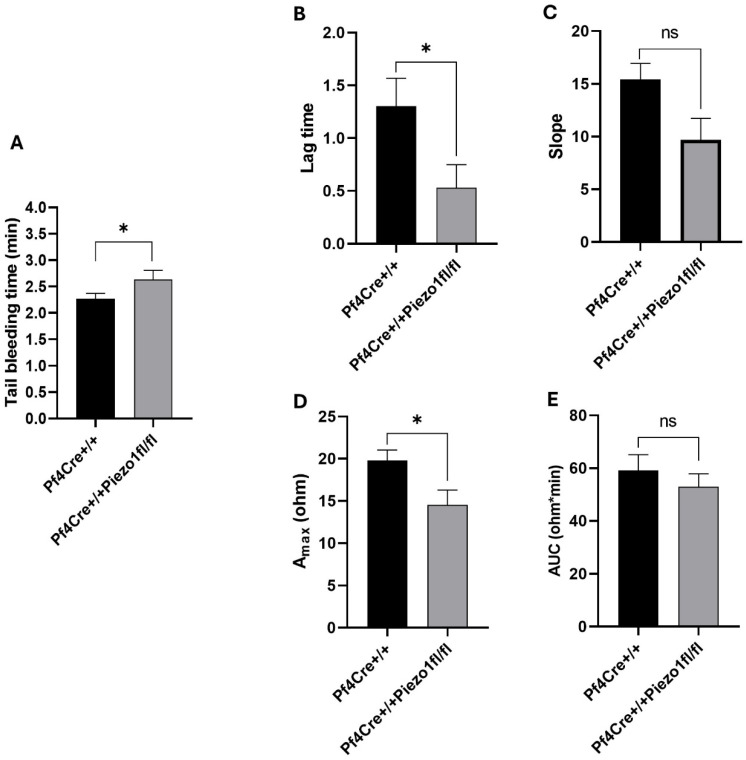
Measurement of tail bleeding time and platelet activation. (**A**) Tail bleeding time was calculated and expressed as minutes to cessation of blood stream from mice tails. Data are expressed as mean ± SE of n = 12 mice ages 12–14 weeks old (both males and females), where * *p* < 0.05 is considered significantly different between PF4-Cre^+/+^ and PF4-Cre^+/+^Piezo1^fl/fl^ mice. (**B**–**E**) Effect of 5 µg/mL of collagen on platelet aggregation measured using Acid Citrate Dextrose (ACD)-anticoagulated whole blood (see the Methods Section). (**B**) lag time, (**C**) slope, (**D**) maximum aggregation (A_max_), and (**E**) area under the curve (AUC). Data are from two independent experiments, each with three mice per genotype. Aggregation parameters are presented as mean ± SD. Statistical analysis was performed using Student’s *t*-test; * *p* < 0.05 was considered significant. ns: denotes no statistical difference.

Peizo1 KO mice exhibited a small but still significant augmentation in average tail bleeding time of 2.63 ± 0.16 min, compared to 2.27 ± 0.09 min measured in PF4-Cre controls (*p* < 0.05) (Figure 4A). In vitro platelet activation studies showed reduced response of platelets to collagen in the Peizo1 KO mice, compared to age and sex matched PF4-Cre control mice (Figure 4B–E). Interestingly, we also observed a tendency for reduction in platelet activation by thrombin (Supplementary Figure S3).

### 3.4. PF4-Cre-Mediated Deletion of Piezo1 Did Not Alter Baseline Levels of Serum Cytokines, but Impacted the Response to Lipopolysaccharide Challenge

**Figure 5:**
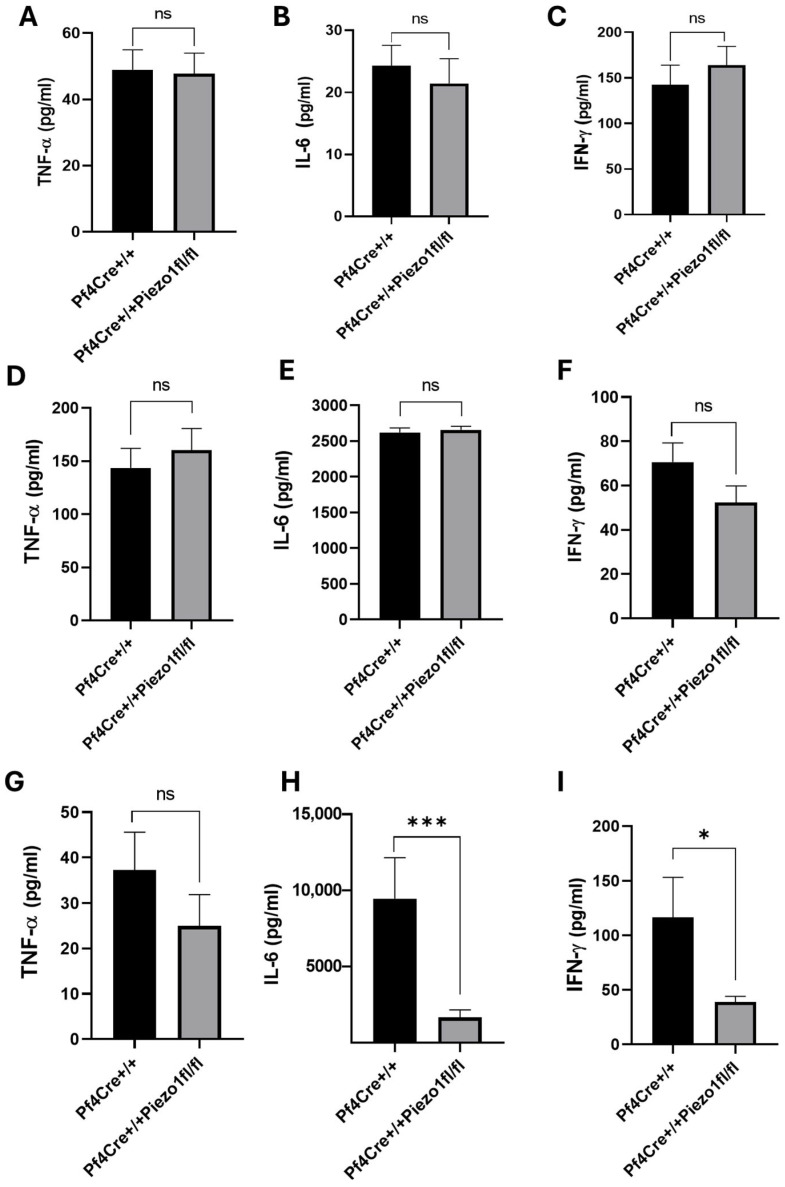
Measurement of serum cytokines in PF4-Cre and Piezo1 KO mice, during baseline and LPS challenge. (**A**–**C**) Proinflammatory and anti-inflammatory cytokines in serum samples of PF4-Cre^+/+^ and PF4-Cre^+/+^Piezo1^fl/fl^ male and female mice at the baseline level. Cytokines levels at 3 hours (**D**–**F**) and 24 hours (**G**–**I**) post-LPS challenge. Data are expressed as mean ± SE (n = 9–14 age-matched mice), where * *p* < 0.05, *** *p* < 0.001 values are considered significantly different between PF4-Cre^+/+^ and PF4-Cre^+/+^Piezo1^fl/fl^ mice. ns: denotes no statistical difference.

Considering the reduced WBC counts measured upon Piezo1 deletion, pro-inflammatory (TNF-α and IFN-γ) and anti-inflammatory (IL-6) cytokines were measured in the serum samples of age–sex-matched PF4-Cre and Piezo1 KO mice. No significant changes in baseline levels of these cytokines were recorded in the two experimental groups (Figure 5A–C). The levels of these cytokines were also measured upon lipopolysaccharide (LPS) challenge, particularly after 3 h (early pathogenesis) and 24 h (late pathogenesis). A Kaplan–Meier survival curve recorded for mice at 3 and 24 h post-LPS treatment showed no death induced by treatment (Supplementary Figure S4). LPS injected intraperitoneally augmented the level of all three cytokines after 3 h by many folds, compared to their baseline values (Figure 5D–F), while no significant differences were recorded among PF4-Cre control and Piezo1 KO groups at that time point (Figure 5D–F).

**Figure 6:**
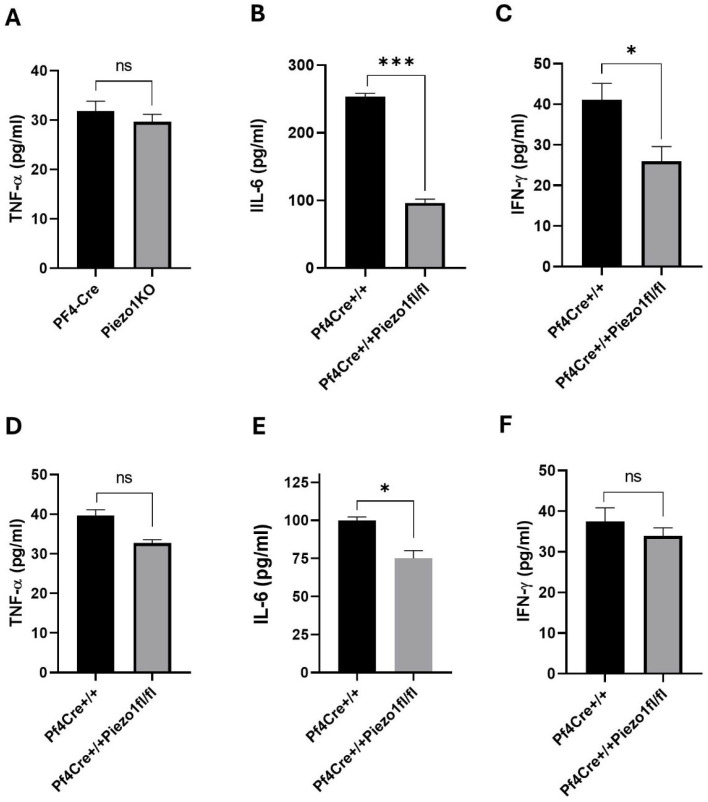
Measurement of cytokines in culture supernatant. (**A**–**C**) Level of cytokines in supernatants of MK-enriched BM cultures (see the Methods Section) derived from PF4-Cre and Piezo1 KO mice. (**D**–**F**) Level of cytokines in supernatants of BSA gradient-purified MKs isolated from PF4-Cre^+/+^ and PF4-Cre^+/+^Piezo1^fl/fl^ mice (n = 8–10). Data are expressed as mean ± SE, where * *p* < 0.05, *** *p* < 0.001 values were considered as significant difference between PF4-Cre^+/+^ and PF4-Cre^+/+^Piezo1^fl/fl^ mice. ns: denotes no statistical difference.

At 24 h post-LPS challenge, the level of TNF-α in Piezo1 KO mice tended to be lower (32.98%, no statistically significant difference) (Figure 5G), as compared to PF4-Cre control. However, upon 24 h of the LPS challenge, the levels of IL-6 and IFN-γ in Piezo1 KO mice were significantly (*p* < 0.001) reduced and normalized to baseline levels, but not in PF4-Cre control mice (Figure 5H–I). These inflammatory cytokines were also measured in the media of cultured bone marrow cells (Figure 6A–C) and of purified MKs (Figure 6D–F), where only the IL-6 level was found to be significantly reduced (Figure 6B,E) upon Piezo1 deletion, as also detected at the mRNA expression level (Supplementary Figure S5). Specifically, a large decrease in IL-6 level was detected in BM cultured cells upon Piezo1 deletion compared to control cultured cells (Figure 6B). The TPO-induced culture includes both low- and high-ploidy, small and larger MKs (as well as mesenchymal cells). On the other hand, the culture of BSA gradient-purified MKs includes primarily large mature cells, potentially explaining the lesser effect of Piezo1 deletion on IL-6 production (Figure 6E). This explanation could also account for a negligible effect of Piezo1 deletion on secreted IFN-γ by cultured purified MKs compared to the BM culture (Figure 6F vs. Figure 6C). To assess effects on Piezo1 activation, PF4-Cre or Piezo1 KO bone marrow cultures were treated for 4 days with vehicle (DMSO) or Piezo1 activator Yoda1 (4 µM). Yoda1 induced a 3–4-fold increase in IL-6 level (but not IFN-γ) in the PF4-Cre culture, and a lower and variable increase in IL-6 (1–2-fold) in Piezo1 KO cultures. This variability and lack of complete ablation of Yoda1 effect in the KO culture could be due to partial knockout in assorted MKs, and/or likely owing to the presence of bone marrow mesenchymal cells in the culture, the latter known to express Piezo1 [24].

## 4. Discussion

Mechanosensors play a crucial role in the interactions of cells within the bone marrow microenvironment, affecting the differentiation of hematopoietic stem cells, which in turn have vital physiological outcomes [3,4]. MK lineage commitment, polyploidy, and proplatelet formation are also regulated by various mechanosensors, where we have recently identified Piezo1 as a regulator of MK development in culture [5,25]. Herein, under in vitro conditions, pharmacological activation of Piezo1 using Yoda1 (a known pharmacological activator) resulted in a significant increase in the number of immature MKs (CD41^+^CD42^−^ cells), along with a lower number of mature MKs (CD41^+^CD42^+^) [13]. Similarly, Demagny et al. studied the mechanical activation of Piezo1 using Yoda1 in cultured MKs and found reduced MK maturation and count [12]. These changes were reverted when *Piezo1* gene expression was suppressed using an shRNA-silencing approach [12]. While these studies suggested a regulatory role of Piezo1 in MK development, they are limited to in vitro findings, especially under pharmacological modulations.

To validate these initial findings and identify the putative role(s) of Piezo1 in BM hematopoiesis, we generated mice with deletion of Piezo1 in the megakaryocytic (MK) lineage and HSC progenitors using PF4-Cre mice cross breed with Piezo1^fl/fl^ mice to yield PF4-Cre^+/+^Piezo1^fl/fl^ homozygous mice (Piezo1 KO). Recently, Piezo1 has been identified as an early regulator of hematopoiesis [26], thus validating our findings concerning the tendency of PF4-Cre-targeted deletion of Piezo1 to affect the level of hematopoietic progenitors. Interestingly, in our study, Piezo1 KO mice did not exhibit any significant difference in the number of mature MKs (CD41^+^CD42^+^) or in the ploidy level compared to PF4-Cre control mice; this discrepancy with in vitro studies might be due to the presence of compensatory mechanism(s) within a more physiological in vivo milieu. In this context, we ruled out a compensatory effect of Piezo2, as its mRNA level was negligible or not detected in repeated assays of MKs derived from control mice or Piezo1 KO mice. Additionally, it is possible that Piezo1 deletion might not have the opposite effect than that of its activation, owing to saturation effects. Piezo1 overexpression and altered megakaryopoiesis have also been reported in primary myelofibrosis (PMF), marked by increased MK number and bone marrow stiffness [13,27]. Herein, altered matrix stiffness might contribute to augmenting Piezo1 expression and, hence, its activation, which could lead to changes in MK maturation [13]. In our current study, bone marrow matrix stiffness was not found to be altered in the Piezo1 KO mice (as per reticulin staining), as compared to their matching controls. Hence, MK maturation and polyploidization remained unaffected upon Piezo1 deletion, in agreement with a previous report [28].

In our study, MK-specific Piezo1 deletion resulted in an approximately 25% increase in platelet count in mice, while there was no parallel increase in MK number to account for this observation. It is possible that reduced vascular adhesion potential of platelets upon Piezo1 deletion manifests in augmenting their level in the circulation. Tail bleeding time estimated in Piezo1 KO mice was slightly higher compared to controls. Also, platelet activation mediated by collagen was found to be reduced in the KO mice, thus validating the measurements of somewhat prolonged tail bleeding in these mice. Our findings are in line with the reported reduced severity of arterial thrombosis and stroke in hypertensive mice upon pharmacological inhibition of Piezo1 [29]. Further, Aglialoro et al. reported that activation of Piezo1 leads to enhanced adhesion of platelets to VCAM1 and fibronectin, thus resulting in integrin activation and thrombus formation [30]. Additionally, in accordance with our findings, a previous study involving pharmacological activation (with Yoda1) or inhibition (via GsMTx-4) of Piezo1 in the MK/platelet lineage reported a stimulating or inhibiting, respectively, effect on platelet activation by collagen and thrombin [31]. These results collectively suggest a pivotal role of platelet Piezo1 in controlling thrombosis, thus validating Piezo1 as a potential drug target.

A relationship between mechanosensitive ion channels and inflammation has been found to be bidirectional, where localized inflammation and mechanical forces (shear stress and tissue stretching) have been reported to activate various ion channels [32]; this activation triggers a series of intracellular events, leading to modulation of expression of inflammatory cytokines through complex signaling cascades [32]. In our study, WBC count was significantly reduced upon Piezo1 deletion in PF4-Cre-mediated Piezo1 KO mice, leading us to evaluate the impact of this state on inflammatory cytokine levels. Detecting a reduced WBC count was not surprising, considering that PF4-Cre targets hematopoietic progenitors, including MPP3 and MPP4 [23]. It is then possible that the lack of Piezo1 affected the number of progenitors differentiating into WBCs, resulting in their reduced level in the circulation. It seems, however, that once MPP4 diverged to fully differentiated WBCs, PF4-Cre was no longer active, as we did not detect a significant reduction in Piezo1 expression in WBCs derived from Piezo1 KO mice. Recent reports are also confirmatory of Piezo1 expression in WBCs, which in turn plays key roles in inflammation [32,33].

Moreover, no significant difference in the levels of TNF-α, IFN-γ, and IL-6 was found between PF4-Cre and Piezo1 KO mice sera at baseline. Surprisingly, LPS-mediated systemic inflammation resulted in reduced levels of IL-6 and IFN-γ in Piezo1 KO mice compared to matching controls (PF4-Cre); this, in turn, is indicative of putative roles of Piezo1 in inflammation, in accordance with other reports [34,35]. Reduced WBC count in the KO mice and/or the deletion of MK Piezo1 could account for the observed reduction in IL-6 or TNF-alpha upon LPS challenge. Indeed, IL-6 is a key cytokine release by WBCs, which in turn influences WBC’s inflammatory behavior [36,37] and the production of inflammatory cytokines [38]. Additionally, IL-6 KO mice have reduced WBC count in response to inflammatory stress [37,38]. As to the contribution of Piezo1 KO MKs to this phenotype, we found that only IL-6 was significantly reduced in these cells when cultured under unstimulated (no LPS) conditions; however, this reduction seems not to have contributed to changes in the mouse plasma level of this cytokine at baseline.

## 5. Conclusions

In conclusion, we identified an unexpected role of Piezo1 in controlling MPP levels, with effects on WBC counts and the inflammatory response to immunogenic challenge. Our results also showed that while Piezo1 is dispensable for MK development and ploidy, it controls MK-specific production of IL-6 cytokine. Finally, we provide direct evidence for the role of Piezo1 in controlling platelet activation response and bleeding time.

## Data Availability

The data supporting the findings of this study are available in the Supplementary Materials.

## Supplementary Materials

The following supporting information can be downloaded at https://www.mdpi.com/article/10.3390/cells14241999/s1. Supplementry Figure S1: (A). Breeding scheme for the generation of PF4-Cre+/+Piezo1fl/fl homozygous transgenic mice. (B). Representative full-scale western blots of Piezo1 expression in PF4-Cre^+/+^ and Piezo1 KO (PF4-Cre^+/+^Piezo1^fl/fl^) BSA gradient-purified megakaryocytes (MKs). (C). Piezo1 mRNA expression in PF4-Cre^+/+^ and PF4-Cre^+/+^Piezo1^fl/fl^ mouse MKs, as measured by qRT-PCR. Supplementry Figure S2: Measurement of Piezo1 mRNA expression in white blood cells isolated from PF4-Cre^+/+^ and Piezo1 KO (PF4-Cre^+/+^Piezo1^fl/fl^) mice. Supplementry Figure S3: Measurement of thrombin-mediated platelet activation. Supplementry Figure S4: Survival kinetic of mice treated with LPS. Supplementry Figure S5: Relative mRNA expression of inflammatory cytokines.
