# Supplementary material for: Piezo1 Mechanosensor Expression in Rare Hematopoietic Cells Controls Systemic Inflammatory Response in Mice
Source: Cells. 2025 Dec 16;14(24):1999. doi: 10.3390/cells14241999 (PMC12732291; doi:10.3390/cells14241999)
Supplement: Supplementary file 1 [file cells-14-01999-s001.zip › cells-3929208-supplementary.pdf]

Supplementry figure 1:

1A.

Piezo1^{floxed} mice (Jackson laboratory- B6.Cg-*Piezo1*$^{tm2.1Apat}$/J)

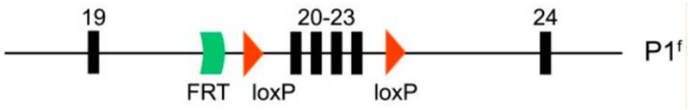

PF4Cre mice (Jackson laboratory- C57BL/6-Tg(Pf4-icre)Q3Rsko/J

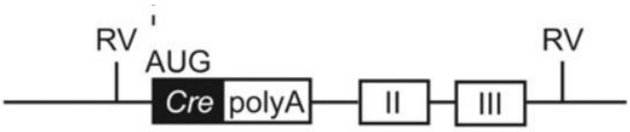

Breeding cycle 1:

Piezo1floxed $^{+/+}$ x PF4Cre$^{+/+}$

↓

100% Piezo1floxed$^{+/-}$PF4Cre$^{+/-}$

Breeding cycle 2:

A. Piezo1floxed$^{+/-}$PF4Cre$^{+/-}$ x Piezo1floxed$^{+/-}$PF4Cre$^{+/-}$

↓

Piezo1floxed$^{+/+}$PF4Cre$^{+/+}$, Piezo1floxed$^{+/+}$PF4Cre$^{+/-}$, Piezo1floxed$^{+/+}$PF4Cre$^{-/-}$
Piezo1floxed$^{+/-}$PF4Cre$^{+/+}$, Piezo1floxed$^{+/-}$PF4Cre$^{+/-}$, Piezo1floxed$^{+/-}$PF4Cre$^{-/-}$
Piezo1floxed$^{-/-}$PF4Cre$^{+/+}$, Piezo1floxed$^{-/-}$PF4Cre$^{+/-}$, Piezo1floxed$^{-/-}$PF4Cre$^{-/-}$

B. Piezo1floxed$^{+/+}$ x Piezo1floxed$^{+/-}$PF4Cre$^{+/-}$

↓

Piezo1floxed$^{+/+}$PF4Cre$^{+/-}$, Piezo1floxed$^{+/+}$PF4Cre$^{-/-}$

Piezo1floxed$^{+/-}$PF4Cre$^{+/-}$, Piezo1floxed$^{+/-}$PF4Cre$^{-/-}$

Breeding cycle 3:

Piezo1floxed$^{+/+}$PF4Cre$^{+/+}$ x Piezo1floxed$^{+/+}$PF4Cre$^{+/+}$

↓

100% Piezo1floxed$^{+/+}$PF4Cre$^{+/+}$

1B.

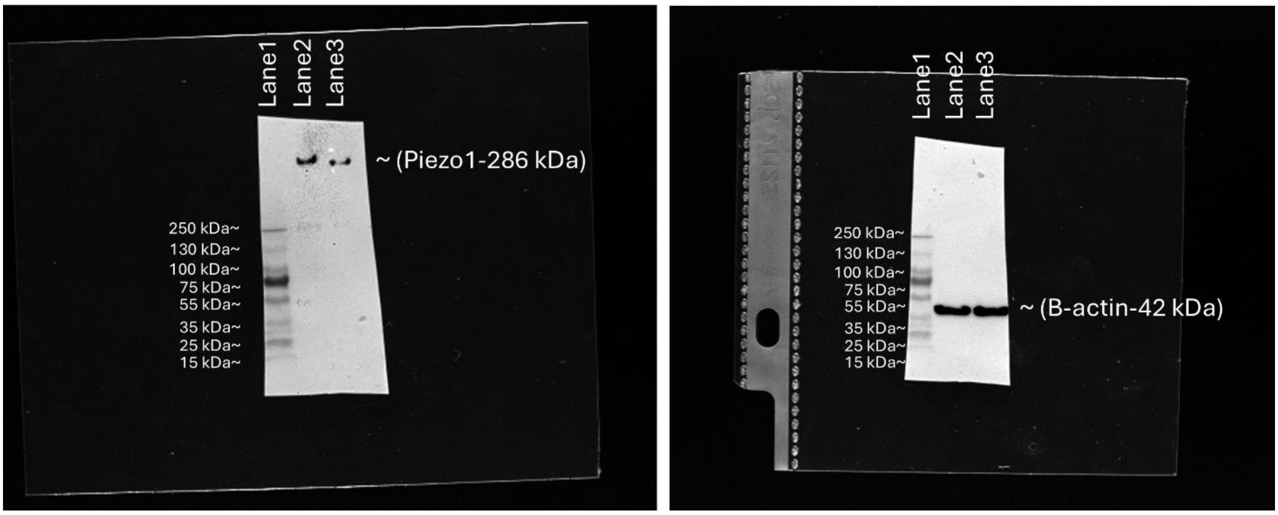

1C.

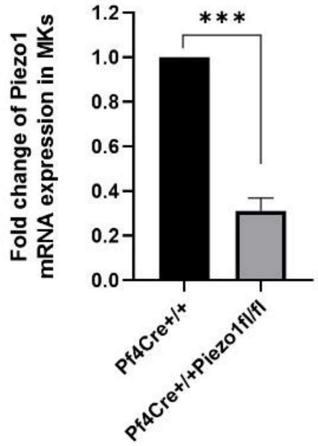

Supplementry figure 1: A. Breeding scheme for the generation of PF4-Cre+/+Piezo1fl/fl homozygous transgenic mice. C57BL/6-Tg(Pf4-icre)Q3Rsko/J mice (Jackson Laboratories), designated as PF4-Cre$^{+/+}$ and B6.Cg-Piezo1tm2.1Apat/J mice (Jackson Laboratories) designated as Piezo1$^{fl/fl}$ were crossbred to obtain PF4-Cre$^{+/+}$Piezo1$^{fl/fl}$ homozygous transgenic mice. **B.** Representative full-scale western blots of Piezo1 expression in PF4-Cre$^{+/+}$ and Piezo1 KO (PF4-Cre$^{+/+}$Piezo1$^{fl/fl}$) BSA gradient-purified megakaryocytes (MKs), with ß-actin antibody used to confirm equal protein loading (n=6, both male and female 12-14 weeks-old mice). In both blots Lane 1 denotes protein markers (pre-stained ladder), Lane 2 denotes PF4-Cre+/+ MK lysate (50µg/lane) and Lane 3 denotes Piezo1 KO (PF4-Cre+/+Piezo1fl/fl) MK lysate (50µg/lane). **C.** Piezo1 mRNA expression in PF4-Cre$^{+/+}$ and PF4-Cre$^{+/+}$Piezo1$^{fl/fl}$ mouse MKs, as measured by qRT-PCR. Data are expressed as mean ± SE (n = 6 age-matched mice), where *** $p < 0.001$ values are considered significantly different between PF4-Cre$^{+/+}$ and PF4-Cre$^{+/+}$Piezo1$^{fl/fl}$ mice.

Supplementry figure 2:

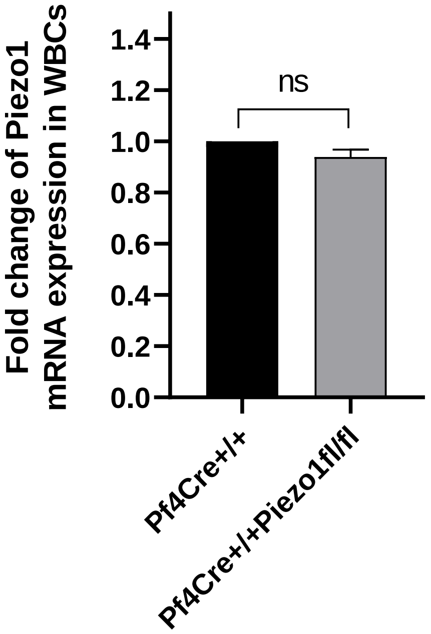

Supplementry figure 2: Measurement of Piezo1 mRNA expression in white blood cells isolated from PF4-Cre$^{+/+}$ and Piezo1 KO (PF4-Cre$^{+/+}$Piezo1$^{fl/fl}$) mice. Piezo1 mRNA (measured via qRT-PCR) expression in white blood cells (WBCs) isolated from blood of PF4-Cre$^{+/+}$ and PF4-Cre$^{+/+}$Piezo1$^{fl/fl}$ mice (n=6, both male and female mice). Values were normalized to 18s ribosomal RNA. Data are expressed as Mean±SEM, where *$p < 0.05$ is considered as statistically significant. ns: denotes no statistical difference.

Supplementry figure 3:

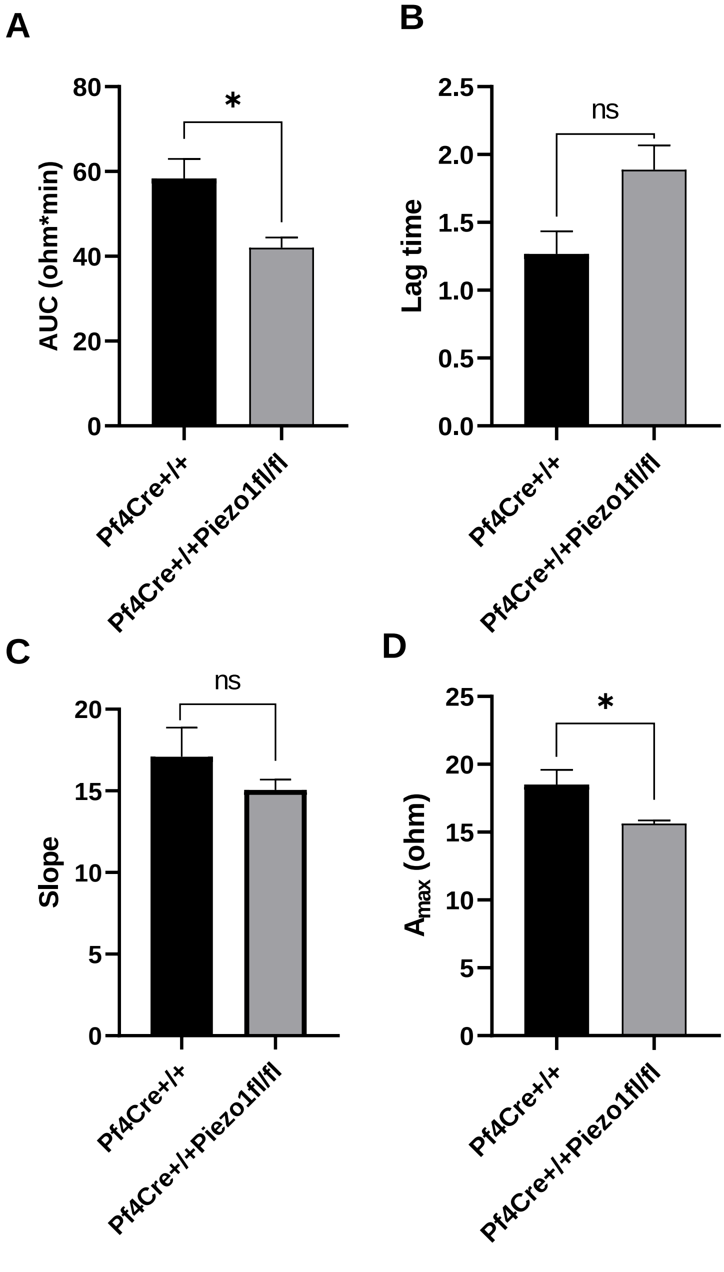

Supplementry figure 3: Measurement of thrombin-mediated platelets activation. (A-D). Effect of 0.025U/mL of thrombin on platelet aggregation, measured using Acid Citrate Dextrose (ACD)-anticoagulated whole blood (see Methods). (A) area under the curve (AUC) (B) lag time, (C) slope, and (D) maximum aggregation (Amax). Data are from two independent experiments. Aggregation parameters are presented as mean± SD. Statistical analysis was performed using Student's t-test; *$p < 0.05$ was considered significant. ns: denotes no statistical difference.

Supplementary Figure 4:

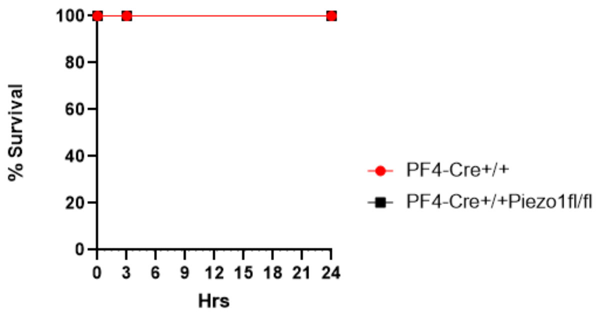

Supplementry figure 4: Survival kinetic of mice treated with LPS. A Kaplan–Meier survival curve of mice at 0, 3 and 24 hrs. n=12 (both male and females mice) for each experiment. Data expressed in term of percent (%) survival.

Supplementary Figure 5:

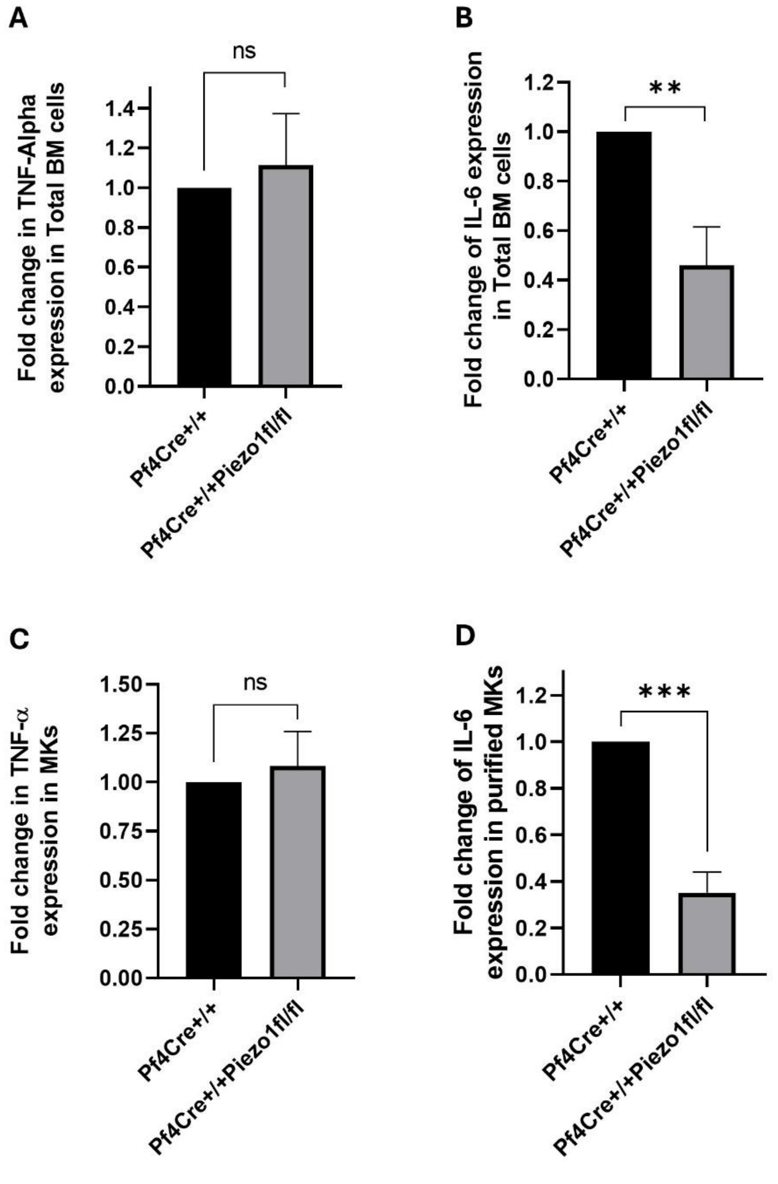

Supplementry figure 5: Relative mRNA expression of inflammatory cytokines. A. TNF-α and **B**. IL-6 mRNA levels were measured in total bone marrow cells isolated from PF4-$Cre^{+/+}$ and PF4-$Cre^{+/+}$$Piezo1^{fl/fl}$ mice (n=5)**.** **C**. TNF-α and **D**. IL-6 mRNA levels were measured in BSA-gradient purified MKs derived from PF4-$Cre^{+/+}$ and PF4-$Cre^{+/+}$$Piezo1^{fl/fl}$ mice and pooled from similar strains. Values were normalized to 18s rRNA. Data are expressed as Mean ± SE of three independent experiments performed separately, where *$p < 0.05$, ***$p < 0.001$ values were considered as significant difference between PF4-$Cre^{+/+}$ and PF4-$Cre^{+/+}$$Piezo1^{fl/fl}$ mice. ns: denotes no statistical difference.
